# A Doubly Robust Approach to Evaluating the Impact of Hospital Length of Stay on Post‐Fracture Recovery in Older Adults With Cognitive Impairment

**DOI:** 10.1002/sim.70768

**Published:** 2026-10-15

**Authors:** Haoyu Ren, Tong Wen, Jason Falvey, Yi Huang, Chixiang Chen

**Affiliations:** ^1^ Department of Mathematics and Statistics University of Maryland, Baltimore County Baltimore Maryland USA; ^2^ Department of Epidemiology and Public Health University of Maryland School of Medicine Baltimore Maryland USA; ^3^ Department of Physical Therapy and Rehabilitation Science University of Maryland School of Medicine Baltimore Maryland USA

**Keywords:** continuous exposure, dementia, doubly robust, hospital length of stay, practical guidance

## Abstract

For older adults diagnosed with Alzheimer's Disease and Related Dementias, recovery outcomes after a hip fracture are often poor, with extended hospital stays and prolonged rehabilitation being common. This paper examines the impact of hospitalization on post‐discharge outcomes, focusing on the differential effects of hospital length of stay on recovery, measured by the number of healthy days spent at home (DAH). Our analysis faces important analytical challenges, including baseline confounding, missing outcome, and the complexity of assessing a continuous exposure. To address these issues, we develop a doubly robust estimator that accommodates a continuous exposure and accounts for outcome missingness and provides practical guidance for end‐users, including diagnostics for conditional density and missing data models and sensitivity analyses for assumption violation. Extensive simulations demonstrate that our method consistently outperforms existing doubly robust method for continuous exposures when outcome data are missing at random. Application to real‐world data suggests that hospital stays longer than 20 days are associated with substantially reduced DAH.

## 1 Introduction

Nearly 300,000 older adults experience a hip fracture each year [1], and recovery after such trauma is often challenging. For those with Alzheimer's Disease and Related Dementias (ADRD), outcomes are particularly poor, often involving extended hospitalizations, prolonged rehabilitation [1, 2], and higher post‐fracture mortality compared to individuals without ADRD. These patients also face greater barriers to restoring their previous level of functioning [3].

Examining hospital length of stay can help clinicians identify individuals at higher risk of poor recovery and thereby support more tailored post‐discharge management strategies to optimize care delivery [4]. This is particularly important for hip fracture patients with ADRD, a population for whom post‐acute recovery trajectories remain insufficiently studied in the clinical literature. In this paper, we apply a modern causal inference framework to examine whether and how hospital length of stay influences post‐discharge recovery, after accounting for baseline factors, to inform discharge planning, followup intensity, and care coordination.

To make valid causal inferences [5, 6, 7], several challenges remain. First, patient‐level and hospital‐level confounding factors at baseline may influence both hospital stay and post‐discharge outcome [1, 8], which will bias the estimation of the true effects. Second, missing outcome data during follow‐up introduce additional complexity to the analysis. Finally, unlike treatment/exposure that can be well categorized into discrete groups, hospital stay lacks established cut‐off points to define clinically meaningful categories. Therefore, we treat hospital length of stay (measured in days) as a continuous exposure.

Currently, few methods can robustly deal with continuous treatment/exposure. Inverse Probability of Treatment Weighting (IPTW) [9, 10, 11, 12, 13, 14] is a widely used approach, where an estimated density function is employed to adjust for confounding factors. Outcome regression is another popular method that models the outcome as a function of both exposure and covariates [15, 16, 17]. A key limitation of both the IPTW and the outcome regression estimators is their reliance on correct model specification. More recently, doubly robust estimators have been proposed [18, 19, 20], which require the correct specification of either the conditional density or the outcome regression, but not necessarily both. Wu et al. [21] developed a matching approach to estimate an average causal exposure‐response function in the context of continuous exposures, which could maintain some degree of robustness against model mis‐specification. However, none of these approaches are designed to handle missing outcome data.

It is important to note that many causal inference methods have been developed to handle missing data. When data is missing completely at random (MCAR), that is, the observation indicator is independent of both observed and unobserved data, complete‐case data analysis remains valid for causal inference. However, when this assumption does not hold, complete‐case data analysis generally leads to biased estimates [22]. In the causal inference literature, a more commonly adopted assumption is missing at random (MAR), under which the observation indicator depends only on observed data. Under the MAR assumption, two primary approaches are widely used to deal with missing data: multiple imputation [23, 24, 25] and inverse of probability weighting (IPW) [9, 10, 11, 12, 13, 14]. Multiple imputation replaces each missing value with several plausible draws from an imputation model conditional on the observed data, generating multiple completed datasets that are analyzed using the pre‐specified substantive model [23, 24]. In practice, this approach is commonly applied to address missing covariates data. On the other hand, IPW–based methods have been proposed to address missingness in both outcomes [26, 27] and covariates [26, 28]. Extensions such as augmented IPW further improve robustness and efficiency by incorporating outcome regression models, yielding doubly robust estimators [29]. In addition, several causal inference approaches have been developed to address missing not at random (MNAR) mechanisms, including selection models [30], pattern‐mixture models [25], and joint modeling strategies [31]. To our knowledge, however, missing data have not been sufficiently studied in the context of continuous treatment/exposure, particularly for the Kennedy's doubly robust framework [18].

In this paper, we develop a new doubly robust method that leverages the causal effect to specifically address key challenges in our analysis and the board in real‐world data analysis, including confounding, missing outcome data, and the complexity of a continuous exposure. The proposed method extends the existing doubly robust estimator [18] to settings with outcomes MAR. It is doubly robust in the sense that it yields valid inference if either (i) the combined model for the conditional density and missingness mechanism is correctly specified, or (ii) the outcome regression model is correctly specified. In addition, we provide practical guidance for end‐users, including diagnostics for conditional density and missing data models as well as sensitivity analyses for assumption violation. To evaluate its utility, we conduct extensive simulation studies to compare its performance with existing methods and apply our approach to Medicare claims data to demonstrate effectiveness in a real‐world setting.

Section 2 introduces the notation used throughout the paper. Section 3 reviews commonly used methods. Section 4 describes our proposed estimator. Section 5 presents a simulation study comparing it with commonly used approaches. Section 6 illustrates the method on real‐world data, and Section 7 concludes with a discussion and directions for future work. Technical proofs and additional numerical results are provided in the Supporting Information.

## 2 Notation and Assumptions

**Notation**. Suppose there are N patients independent and identically distributed. For each patient i from 1 to N, let $A_{i}$ be a continuous treatment/exposure variable of our interest. For example, this variable can be the dosage of medication or hours of training in real applications. Let ${\text{X}}_{i}$ be the vector of confounders, which could include baseline patient‐level factors, such as sex, age, race, and so forth. To formally define causal estimand, we adopt the counterfactual framework. Under the Stable Unit Treatment Value Assumption [5], let $Y_{i}^{a}$ be the potential outcome if the i‐th patient were to receive treatment/exposure a. In the remainder of the article, we focus on the causal parameter $\beta_{0}$ defined by

(1) $$𝔼\left\{\eta (A)(Y^{A}-\eta {\left(A\right)}^{⊤}\beta_{0})\right\}=\text{0}.$$

The estimating equation arises from projecting $Y^{A}$ onto the linear space spanned by η(A). We extended the corresponding doubly robust framework to settings with missing outcomes under a MAR assumption [19]. Rather than directly estimating $E(Y^{a})$, which is generally a complicated non‐linear functional of a, the $\eta {\left(a\right)}^{⊤}\beta_{0}$ (a working function) serves as a tractable approximation of $E(Y^{a})$, enabling more interpretable and favorable statistical inference [19]. Specifically, this working function could in principle be mis‐specified, but the target parameter $\beta_{0}$ is defined as the reasonable approximation of the true curve within the span of η(A). In other words, we are imposing a interpretable parametric working model that captures the main features of the true relationship. Similar idea has been adopted in studies of evaluating conditional average causal effects [19].

Since the Equation (1) involves potential outcomes that are not fully observable in practice, identifying the parameter $\beta_{0}$ requires introducing certain nuissance functions and identification assumptions. Specifically, denote the true mean outcome given covariates and treatment/exposure by $\mu_{0}(a,\text{x})=E(Y|\text{X}=\text{x},A=a)$, the true conditional treatment/exposure density given covariates by $s_{0}(a|\text{x})=\partial P(A\leq a|\text{X}=\text{x})/\partial a$, and the true marginal treatment/exposure density by $\omega_{0}(a)=\partial P(A\leq a)/\partial a$. Moreover, let R be the indicator function with R=1 if the outcome is observed, and R=0 if it is missing, and let $\pi_{0}(A,\text{X})=P(R=1|A,\text{X})$ be the true probability of observing the outcome given all the covariates and treatment/exposure. Throughout the remainder of the article, we use μ(a,x), s(a|x), ω(a), and π(A,X) to denote the corresponding specified models, respectively. The identification assumptions are summarized below:

Assumption 1 (Positivity) For every combination of covariates and exposure status, there is a positive probability of receiving each exposure level and observing outcomes, that is, $s_{0}(a|\text{x})>0$, and $0<\pi_{0}(a,\text{x})<1$, for any possible a and x values.

Assumption 2 (Ignorability) Conditional on the measured covariates X, the treatment/exposure assignment A is statistically independent of every potential outcome $Y^{a}$, that is, $Y^{a}⊥A|\text{X}$.

Assumption 3 (MAR) The missing data mechanism follows MAR assumption, that is, the probability that an observation does not depend on the unobserved (missing) values themselves, conditional on the observed covariates and treatment/exposure: R╨Y|X,A.

The first two assumptions are widely adopted in the causal inference literature [18, 19, 20, 32], while the last is common in the missing data literature with MAR [33, 34]. In our case study, we collect a rich set of baseline variables, including pre‐fracture days at home (DAH), which are strong prognostic of both post‐fracture DAH outcome metric and the missingness mechanism [1, 8]. Therefore, Assumption 3 is plausible in this context.

## 3 Some Commonly Used Methods for a Continuous Treatment/Exposure

**Inverse probability of treatment weighting (IPTW).** IPTW is one of the most widely used methods in causal inference to adjust for confounding when estimating the effect of a binary treatment/exposure. However, when the treatment/exposure variable A=a is continuous (e.g., drug dosage), the standard IPTW approach needs to be modified. In the continuous treatment/exposure setting, the probability P(A|X) is replaced with a conditional density function, since A is no longer binary. Rather than modeling P(A=1|X), we model and estimate the propensity density function $s_{0}(A=a|\text{X})$, which represents the conditional density of receiving treatment/exposure level A given covariates X. Some parametric likelihood approach can be used to estimate the model parameters. After estimation, the stabilized weight is given by $\hat{\omega }(A)/ŝ(A|\text{X})$ [11, 13, 35], where $\hat{\omega }(A)$ is the estimated marginal density of the treatment/exposure. These weights are then used to construct a pseudo‐population in which the treatment/exposure is independent of the covariates, enabling consistent estimation of the causal effect [10]. It is important to note that the IPTW is valid if the propensity density model is correctly specified and can be biased under MAR if complete‐case data are used. To accommodate the context of MAR, additional IPW, $1/\hat{\pi }(A,\text{X})$, can be incorporated to obtain a consistent estimator [10].

**Outcome regression (OR).** On the other hand, outcome regression is a popular approach for estimating treatment/exposure effects by modeling the outcome as a function of the treatment/exposure and covariates. Formally, we model and estimate the conditional expectation of the outcome Y given covariates and the continuous exposure $\mu_{0}(A,\text{X})$ using flexible regression techniques, such as generalized linear models [15]. Once μ is estimated, we can estimate marginal outcome mean function using the rule of 𝔼[Y|A=a]=E(E(Y|A=a,X)|A=a), which represents the expected outcome if all individuals received treatment/exposure level a. This allows us to investigate how varying treatment/exposure intensity influences the outcome. This framework yields consistent estimators if the outcome regression model is correctly specified under MAR.

**Doubly robust (** ${\text{DR}}_{cc}$ **) estimation.** The previous two methods rely on correct specification of either the propensity score model or the outcome regression model, which may be difficult to guarantee in practice. Kennedy et al. [18] proposed a doubly robust estimator, which remains consistent if either one of two models is correctly specified, but not necessarily both, thus providing some robustness against model misspecification. Specifically, they constructed the following pseudo outcome for each individual i:

(2) $$Y^{*}(\tilde{\xi })=\frac{Y-\mu (A,\text{X})}{s(A|\text{X})}\omega (A)+\int \mu (A,\text{x})dF(\text{x}),$$

where $\tilde{\xi }=\{s,\omega ,\mu \}$ is defined before and can be estimated by certain parametric regression methods. Here, the term “pseudo outcome” refers to a technical, intermediate quantity constructed from observed data to facilitate estimation. After the calculation of the pseudo outcome $Y^{*}(\tilde{\xi })$ with all estimated nuisance models and numerically estimated integral in the Equation (2) [18], $E(Y^{a})$ can be estimated using kernel regression [18] or project‐based [19] approaches. Noted that this doubly robust estimator using complete‐case data can be inconsistent under MAR.

## 4 The Proposed Method

As described in Section 3, the existing ${\text{DR}}_{cc}$ estimator [18] using complete‐case data could be inconsistent, particularly when missing data mechanism follows MAR. In this section, we describe a doubly robust approach to conveniently estimate $\beta_{0}$ in the presence of missing outcome. Instead of using the pseudo outcome described in (2), we consider the revised version:

(3) $$Y^{*}(\xi )=\frac{R}{\pi (A,\text{X})}\left[\frac{Y-\mu (A,\text{X})}{s(A|\text{X})}\omega (A)\right]+\int \mu (A,\text{x})dF(\text{x}),$$

where ξ={π,s,ω,μ} is the nuisance functions of our proposed estimation. All notations have been defined in previous sections. To estimate the integral in the Equation (3), we use the leave‐one‐out method [19], which could avoid self‐influence/overfitting. The difference between the proposed method and the Kennedy's doubly robust estimation [18] is that we add an additional weight function to the first part of Equation (3). The intuitive rationale is that inverse probability of weight will address the biased sampling issue in the first part of the Equation (3) so that missing data is no longer introducing bias to IPTW component. However, we do not add this weight to the second part of the Equation (3), in order to promote doubly robust property of the resulting estimator.

After computing $Y^{*}(\hat{\xi })$, where $\hat{\xi }$ contain the estimators of the parameters in ξ, we regress it onto the linear space spanned by η(A) to obtain the $\hat{\beta }$. The following theorem summarizes the asymptotic property of $\hat{\beta }$. The pseudo algorithm for the proposed estimation is summarized in Algorithm 1.

ALGORITHM 1 **Proposed algorithm.**

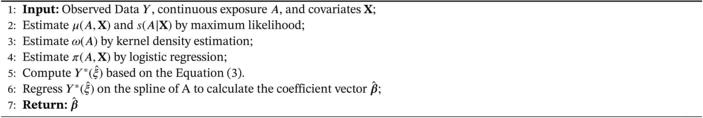

Theorem 1 (Consistency) *Under the Assumptions* 1 *to* 3 *and regularity conditions described in Section S*1 *of the* *Supporting Information*, *we have that* $\hat{\beta }$ *is the consistent estimator of* $\beta_{0}$ *satisfying*

$$\left\|\hat{\beta }-\beta_{0}\right\|\;{≲}_{P}\;\sqrt{\frac{d}{N}}+\left[\left(\sqrt{\frac{d}{N}}\;l_{d}r_{d}\right)\wedge \left(\epsilon_{d}r_{d}/\sqrt{N}\right)\right],$$

*provided that*(*i*) $\mu =\mu_{0}$ *or* (*ii*) $\pi =\pi_{0}\;\&\;s=s_{0}$ *holds, but not necessary both. Functions* $\mu_{0},\pi_{0},s_{0}$ *represent true nuisance functions, the* ‖·‖ *represent the* $\ell_{2}$ *norm of a vector, and the notations* d, $l_{d}$, $r_{d}$ *, and* $\epsilon_{d}$ *are defined in Section* S1 *of the* *Supporting Information*.

We highlight two remarks. First, we recommend using a bootstrap procedure for statistical inference, since the asymptotic variance for a doubly robust estimator is complicated. Bootstrap is implementation‐robust and often yields reliable finite‐sample performance when nuisance parameters are estimated and then plugged into the proposed estimator. Alternative variance estimation methods can also be adapted when parametric modeling approaches are used to estimate nuisance functions [36]. Second, although the proposed estimator is doubly robust, in practice it may still yield biased estimates if all models are mis‐specified. Therefore, we provide detailed guidelines below to inform the best practices for applying the method.

**Model diagnosis**. To evaluate if the propensity density model and missing data model are appropriate to use, it is common to evaluate if covariates are balanced based on the reweighted samples. Specifically, for the propensity density model, we first compute reweighted samples using estimated $\hat{\omega }(A)/ŝ(A|X)$ as weights, and then evaluate the weighted correlation coefficient between the treatment/exposure and each covariate. Correlation coefficients close to zero indicate good balance across treatment/exposure levels [37]. On the other hand, a common strategy for assessing the missing data model is to examine whether the distributions of baseline covariates are well balanced between two groups, the entire cohort and the subcohort without missing, using weighted samples ($R/\hat{\pi }(A,X)$). Specifically, we visualize the standardized mean difference (SMD) for each covariate using a love plot. Following established guidelines [38], an SMD value below 0.1 may indicate adequate balance.

**Sensitivity analysis for departures from MAR.** To assess sensitivity to a possible violation of MAR assumption, we consider an unmeasured factor U that may be associated with both the outcome and its probability of being observed. Let $\pi_{0}(X_{i},A_{i},U_{i})=P(R_{i}=1|X_{i},A_{i},U_{i})$ denote the underlying conditional probability, and let $\mu_{0}(X_{i},A_{i},U_{i})=E(Y_{i}|X_{i},A_{i},U_{i})$ denote the corresponding conditional outcome mean. We define the departures from the observed‐data models as $\xi_{\pi ,i}=\mathrm{logit}\{\pi_{0}(X_{i},A_{i},U_{i})\}-\mathrm{logit}\{\pi_{0}(X_{i},A_{i})\},$ and $\xi_{\mu ,i}=\mu_{0}(X_{i},A_{i},U_{i})-\mu_{0}(X_{i},A_{i}).$ Given the unobserved nature of U, we cannot observe or estimate $\xi_{\pi ,i}$ and $\xi_{\mu ,i}$ using the observed data. Alternatively, we propose a simulation‐based sensitivity analysis: we generate a shared subject‐specific offset for the observation and outcome models, that is, $\xi_{\pi ,i}(\psi )=\xi_{\mu ,i}(\psi )=\psi s_{Y}{Ũ}_{i},$ where ${Ũ}_{i}∼N(0,1)$, $s_{Y}$ is the empirical standard deviation of the observed outcome, and ψ is a non‐negative‐valued sensitivity parameter quantifying the level of MAR departure, with larger values representing stronger departures from MAR. When ψ=0, the shared offset is zero and the analysis reduces to the primary MAR analysis.

For each sensitivity level, we generate subject‐specific offsets and refit both the observation and outcome models using these offsets. This step is repeated multiple times, with the corresponding equation calculated for each generated dateset. Finally, the estimating equations are averaged across generated datasets to solve for the target parameter. This repeated generation procedure is designed to mitigate the impact of any single realization of the generated variable. Note that this sensitivity analysis relies on a specific shared perturbation to both outcome and missingness models. The stability observed under this setting does not necessarily guarantee similar performance under general MNAR assumption (e.g., ${Ũ}_{i}∼N(f({\text{X}}_{i},U_{i}),1)$ for some function f(·)). Nevertheless, we consider this analysis strategy a natural approach to exploring the potential impact of MNAR.

## 5 Simulation

### 5.1 Data Generation

We generated a mixture of continuous and binary covariates. Specifically, we simulated ten covariates X, five continuous variables followed by a multivariate normal distribution with mean zeros and variance‐covariance matrix Σ and five categorical variables followed by a independent multivariate binomial distribution with means 0.5 and variance matrix ρ. Details for ρ and Σ matrices can be found in the Section S3 of the Supporting Information.

Moreover, we generated a continuous exposure A from a Beta distribution, Beta(α,α), where $\alpha =exp({\text{b}}^{⊤}\text{X})+1$ with $\text{b}={\left(0.3,-0.4,-0.4,0.4,0.1,0.3,-0.8,-0.4,0.1,0.3\right)}^{⊤}$. We generated the outcome by the model $Y(A)=\mu_{0}(A,\text{X})+e$, where $\mu_{0}=\gamma^{⊤}\text{X}+A*(1-0.5\text{X})$ with 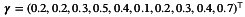 and a demeaned residual e∼Gamma(1,1)−1. Rather than drawing the error e from a normal distribution, we used a gamma distribution to induce skewness and heavier tails, yielding more realistic noise.

We also generated a variable R to indicate if the outcome was observed for each patient, where R=1 denotes an observed value and R=0 denotes a missing value. This variable followed a Bernoulli distribution with a success probability $l\{{\text{c}}^{⊤}\text{X}+0.5A\}$, where l is a logit link function and $\text{c}={\left(0.2,0.3,0.7,0.6,9.5,0.9,0.4,1.5,0.3,0.1\right)}^{⊤}$. The values 0 and −1.3 were selected so that the missing outcome rates were approximately 20% and 40%, respectively. Regarding $\eta {\left(A\right)}^{⊤}\beta_{0}$, we considered a varying coefficient model in which η(A) based on a cubic spline basis of A. The true value of $\beta_{0}$ value was obtained via computer simulation (see Section S3 of the Supporting Information for details). To evaluate the doubly robust property of the proposed estimator, we additionally examined settings in which the nuisance models were misspecified. In particular, the misspecified outcome regression model ${\hat{\mu }}_{mis}(A,X_{1})$ included only A and $X_{1}$, the misspecified treatment/exposure model ${\hat{\pi }}_{mis}(A)$ included only A, and the misspecified sampling model ${ŝ}_{mis}(A|X_{1},X_{2},X_{3})$ included only $X_{1}$, $X_{2}$, and $X_{3}$.

We compared the performance of the proposed approach with three adapted methods. Specifically, for the estimator from the work of Kennedy's doubly robust estimator, we constructed the pseudo outcome in Equation (2), using only the observed data and then regressed it on the cubic spline basis of A, that is, η(A). We refer to the resulting estimator as ${\hat{\beta }}_{{DR}_{cc}}$. Note that this estimator only considered the complete‐case setting. Similarly, we obtained the IPTW‐based estimator by weighting the observed outcome with the estiamted stabilized weight and IPW, that is, $R\hat{\omega }(A)/\left\{\hat{\pi }(A,\text{X})ŝ(A|\text{X})\right\}$ described in Section 3. The resulting pseudo outcome was then regressed on η(A), yielding ${\hat{\beta }}_{IPTW}$, which accommodated missing outcomes. Finally, we obtained the outcome regression‐based estimator using the leave‐one‐out approach to estimate $\int \hat{\mu }(A,\text{x})dF(\text{x})$ as the pseudo outcome and regressing it on η(A), yielding ${\hat{\beta }}_{OR}$. Because the baseline covariates were assumed to be fully observed, the outcome regression‐based estimator was computed using all available data after fitting the model. For fair comparison, the specifications of all nuisance models were kept consistent with those used in the proposed method. The evaluation was conducted using 500 simulated datasets with sample size N=1000 and 3000, and missing rates of 20% and 40%. For each method, we calculated the root‐mean‐squared error (RMSE) as $\sqrt{\text{mean}{\left[\eta {\left(A\right)}^{⊤}*\beta_{0}-\eta {\left(A\right)}^{⊤}*\hat{\beta }\right]}^{2}}$ to assess performance in terms of estimation consistency. Here, $\hat{\beta }$ denotes a generic estimator under study. For statistical inference check, we reported four individual parameters associated with the spline basis of A (with degree equal to three) and only focused on the proposed estimator because bootstrap procedures for the commonly used approaches have been well studied.

### 5.2 Result Evaluation and Interpretation

**Bias**. The estimation bias under a 20% missing rate are summarized in Figure 1. When all models were correctly specified, the ${\text{DR}}_{cc}$ estimator exhibited slight bias, whereas the other three showed negligible bias. This is expected, as the ${\text{DR}}_{cc}$ estimator from [18] does not account for missing data. In contrast, when all nuisance functions were mis‐specified, all methods produced biased estimates; however, the proposed estimator (${\text{DR}}_{mar}$) still achieved the smallest RMSE. Furthermore, the proposed estimator consistently yielded unbiased estimates in the following scenarios: (a) both the propensity score π(·) and conditional density s(·) mis‐specified, (b) only π(·) mis‐specified, and (c) only s(·) mis‐specified. The other three estimators, by contrast, were biased in at least one of these settings. When missing rate was increased to 40% (Figure 2), larger estimation biases were observed for the existing methods when one model was mis‐specified; in contrast, the proposed method continued to yield consistently small bias in most scenarios. These results demonstrate the robustness of the proposed estimator. Similar patterns were observed under a sample size of 1000 (Figures S1 and S2 in the Supporting Information).

**FIGURE 1:**
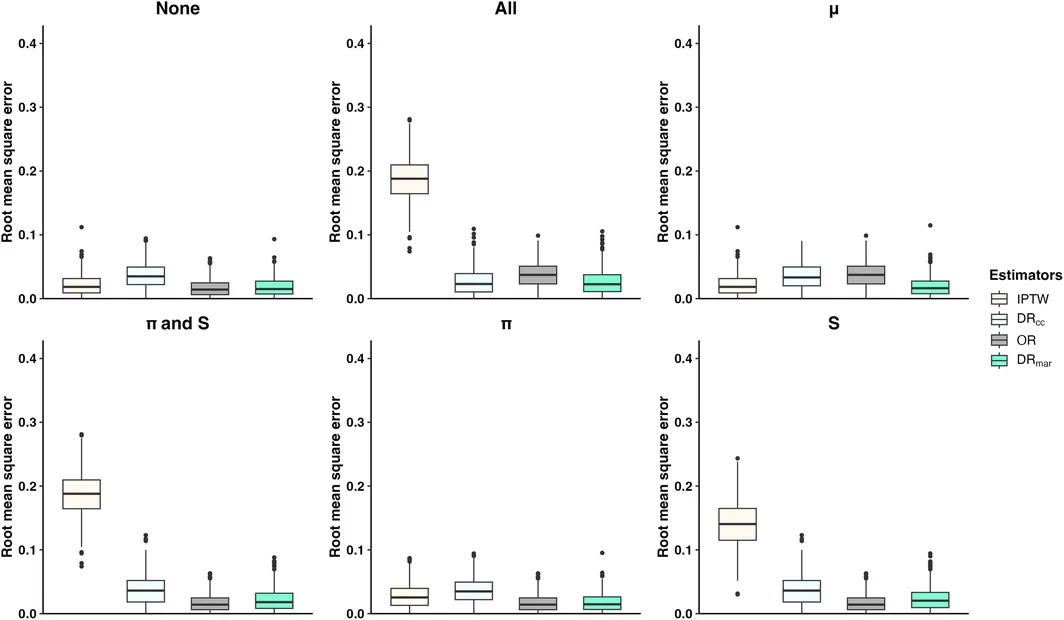
Evaluating performance of different estimators under settings with different misspecified models, sample size of 3000, and missing rate 20%.

**FIGURE 2:**
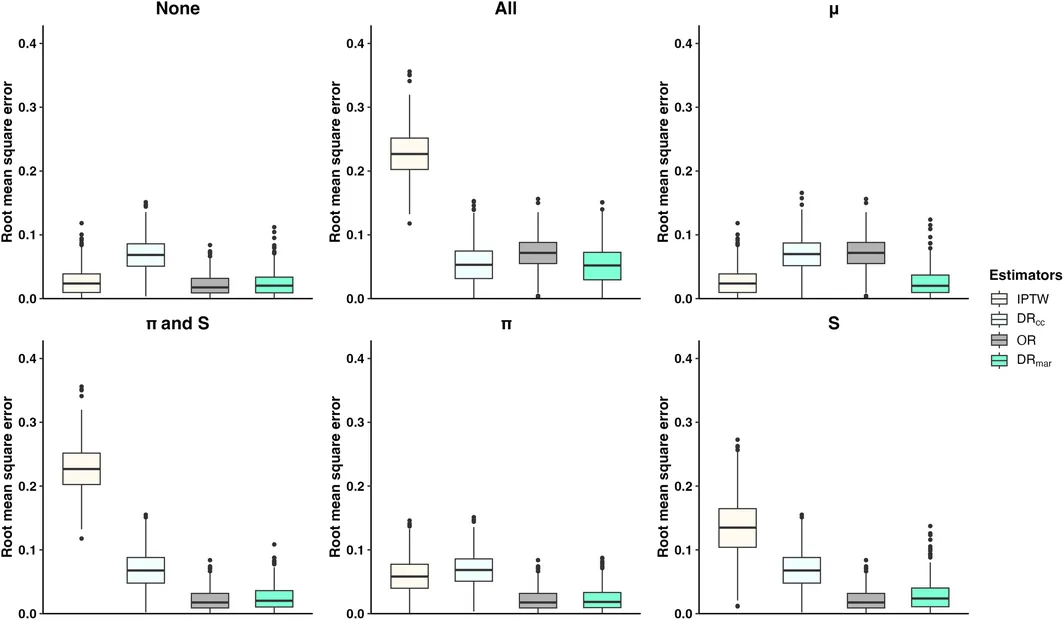
Evaluating performance of different estimators under settings with different misspecified models, sample size of 3000, and missing rate 40%.

**Statistical inference**. For estimation variance, the OR estimator had the smallest variance, while the IPTW estimator showed the largest variability across all scenarios (Figures 1 and 2). The proposed estimator generally led to smaller variance than the ${\text{DR}}_{cc}$ estimator. Moreover, the bootstrap‐based standard errors (BSE, 50 resamples) of the proposed estimator closely matched the Monte Carlo standard deviation, and the resulting coverage probabilities of the bootstrap confidence internals were close to the nominal level 95% (Table 1). These results support the use of bootstrap‐based inference in practice. Tables for other scenarios could be found in Tables S1, S2, and S3 in the Supporting Information.

**TABLE 1:** Evaluation of bootstrap standard error (BSE) and coverage probability (CP) under different settings of mis‐specified models, sample size of 3000, and missing rate 40%.

	None		μ		S		π		π & S	
	BSE(MCSD)	CP	BSE(MCSD)	CP	BSE(MCSD)	CP	BSE(MCSD)	CP	BSE (MCSD)	CP
$\beta_{1}$	21.5 (24.2)	95	28.0 (33.1)	94	38.5 (42.1)	95	20.1 (24.4)	93	32.7 (34.8)	93
$\beta_{2}$	34.8 (37.1)	95	43.7 (47.7)	94	53.6 (55.1)	95	31.8 (36.5)	93	46.0 (47.7)	93
$\beta_{3}$	34.7 (37.0)	94	40.5 (43.4)	94	51.5 (50.8)	94	32.0 (35.5)	94	45.0 (45.3)	93
$\beta_{4}$	21.4 (24.1)	95	23.0 (25.6)	95	34.7 (34.7)	95	20.5 (22.5)	96	30.9 (30.2)	95

*Note:* All values were multiplied by 100.

Moreover, we conducted two sensitivity analyses. One was to evaluate the performance under 2000 Monte Carlo iterations. As the results based on 500 Monte Carlo iterations (Figure S2 in the Supporting Information) were very similar to those based on 2000 iterations (Figure S3 in the Supporting Information), 500 Monte Carlo runs appeared sufficient to provide stable estimates of bias and coverage probability. The other was to evaluate the performance under 500 bootstrap samples. Notably, increasing the number of bootstrap samples from 50 to 500 produced similar inference results, suggesting that 50 bootstrap samples (Table S3 in the Supporting Information) might be adequate for estimating standard errors and coverage probabilities in this simulation study (Table S5 in the Supporting Information).

## 6 Application

**Data description.** We aimed to examine whether and how hospital length of stay after a hip fracture impacts post‐discharge recovery. To achieve this, we analyzed Medicare fee‐for‐service claims data, including a 5% random samples of national beneficiaries from 2010 to 2016 and a 20% random samples from 2017 and 2018. The study population consisted of beneficiaries aged 65 years and older who were hospitalized with hip fractures, identified through the ICD‐9 and ICD‐10 codes for femoral neck or intertrochanteric fractures, excluding pelvic rim or acetabulum fractures [39]. Post‐discharge recovery was measured using “days spent at home” (DAH), a claim‐based metric reflecting how older adults live in their own home and community safely, independently, and comfortably [40]. We defined the outcome as the average number of DAH within the six months after hospital discharge, calculated by subtracting days spent in hospitals, nursing homes, or rehabilitation facilities from 180 days; patients who died during following‐up were coded as missing. Thus, deaths or loss to follow‐up within six‐month period were treated as censored outcomes. We further restricted the analyses to survivors with hospital stays of less than 30 days, as those with longer stays or death during hospitalization represent a distinct subgroup [41, 42]. After data processing, the final cohort contained a total of 21 676 patients, where 23.3% patients had missing outcomes.

To account for baseline factors as potential confounders, we included patient‐level characteristics, such as sex, race, age group, and surgery type for the hip fracture. We also included the number of pre‐fracture DAH during the six months prior to admission date as a proxy for baseline health, along with basic hospital characteristics such as bed size to reflect differences in capacity between small and large hospitals. A directed acyclic graph summarizing the relationships among confounders, the exposure, and the outcome is presented in Figure S4 of the Supporting Information.

**Competing methods and nuisance model estimation** We applied the proposed estimator to the cohort described above and compared its performance with three existing methods: the doubly robust estimator from Kennedy et al. [18] (${\text{DR}}_{cc}$), IPTW, and OR approach. Parametric approaches were used to estimate the nuisance models. Specifically, we used a generalized linear model (GLM) to estimate π(A,X) with both exposure and confounders, a parametric method (GAMLSS) to estimate the conditional density of the exposure given confounders; and a linear model with a cubic B‐spline for the exposure and its interactions with all confounders to estimate the conditional outcome mean using both exposure and confounders. Observations with missing values were excluded for the ${\text{DR}}_{cc}$ estimator [18]. To account for missing outcomes, the weighted IPTW estimator described in Sections 3 and 5 was used. In addition, point‐wise 95% confidence intervals were constructed using the bootstrap standard error (BSE), with limits given by estimate ±1.96×BSE.

**Model diagnose** As described in Section 4, we evaluated the performance of the estimated missing data model and the conditional density function. In Figure 3A, some covariates showed absolute SMD values greater than 0.05, whereas the weighted covariate means were within 0.02 and centered around zero. These results suggest that the estimated missing data model appropriately balances covariates between the missing and observed data. Moreover, Figure 3B shows that correlations in the weighted samples were much closer to zero compared with those in the unweighted samples. This result suggests that the conditional density model might be appropriate.

**FIGURE 3:**
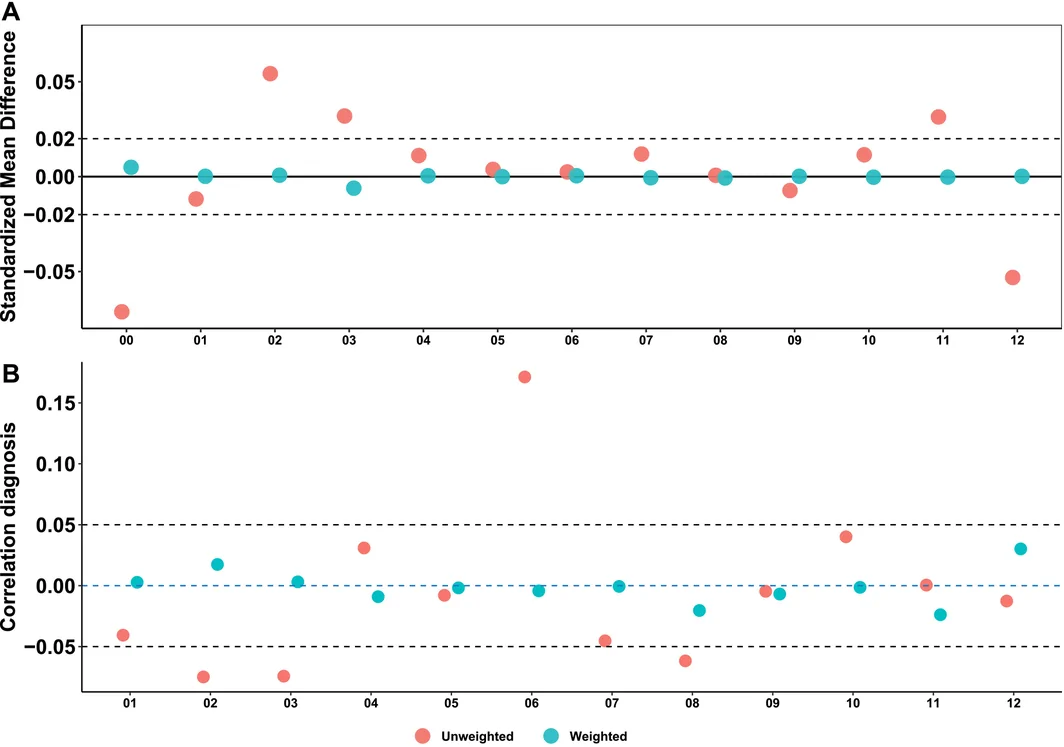
Model diagnosis. (A) Love plot for missing model; (B) weighted correlation evaluation for conditional density model. (The dictionary for the covariate names could be found in the Section S4 in the Supporting Information).

**Result** The main analysis results are summarized in Figure 4. Across all methods, we observed a negative association between hospital length of stay and post‐discharge DAH. This finding is clinically plausible, as longer stays generally reflect poorer health status, greater illness severity, or complications, all of which require longer recovery and often necessitate post‐acute care, thereby reducing time spent at home after discharge. Using the proposed method, we further observed a plateau between 10 and 20 days of hospital stay, during which days at home remained relatively stable; beyond 20 days, days at home decreased dramatically. The ${\text{DR}}_{cc}$ [18], the outcome regression with cubic splines showed a similar non‐linear pattern. IPTW yielded a similar pattern to that of the proposed method, however, with the estimated DAH ranging from 0 to 90 days.

**FIGURE 4:**
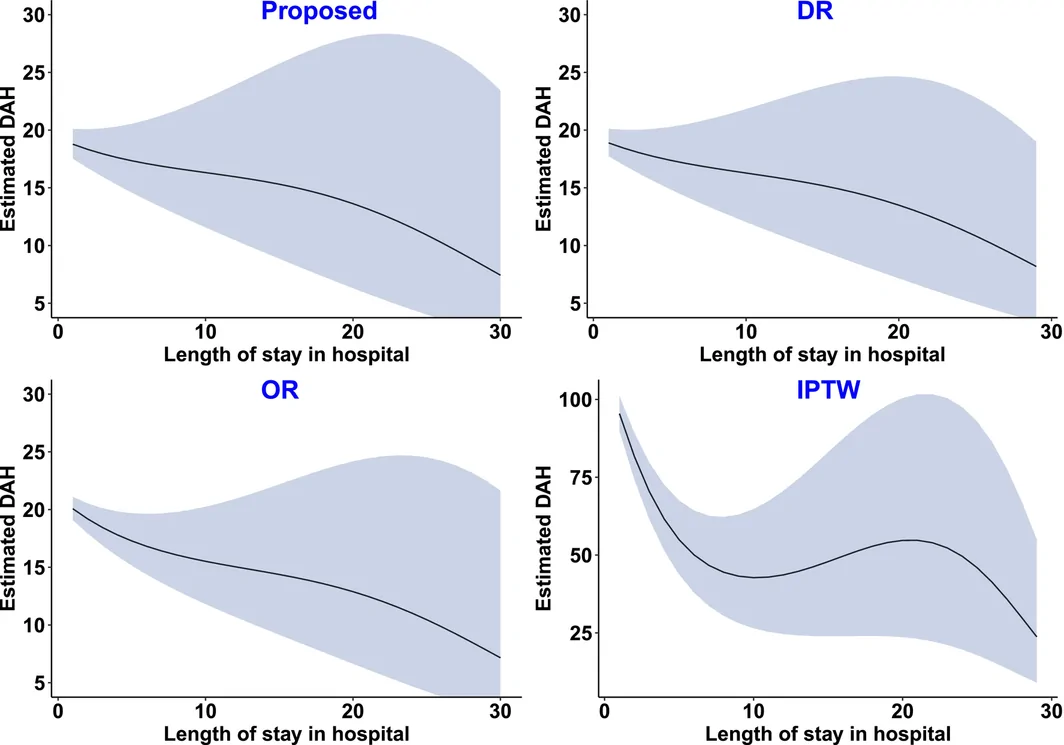
Estimated DAH associated with hospital length of stay. ${\text{DR}}_{cc}$: the doubly robust estimator proposed by Kennedy [18]; OR: the outcome regression estimator; IPTW: the inverse probability of treatment weighting estimator.

Most missing outcomes were due to death among this vulnerable population (around 98%), whereas dis‐enrollment within six months after hospital discharge was uncommon among this population. This raises concerns about the plausibility of the MAR assumption and represents an important limitation of applying the proposed method in this setting. We therefore conducted a sensitivity analysis to assess the potential impact of departures from MAR, with the results summarized in Figure 5. In our case study, we considered five sensitivity levels: ψ∈{0,0.05,0.10,0.15,0.20}. We set the variability of the latent factor to be proportional to the variability of the observed outcomes and selected sensitivity levels ranging from 0% to 20% of the observed outcome variability as an exploratory range. These settings were intended to specify the magnitude of the unmeasured confounders, with 0% corresponding to the primary MAR analysis and 20% corresponding to moderate unmeasured confounding. Across these sensitivity levels, the estimated curves retained the same decreasing pattern. Increasing the variance contribution of the latent factor resulted in a small upward shift in the estimated curve, but the differences among the sensitivity scenarios were modest. We used this analysis to identify potential sensitivity to the specified departures from MAR: substantial changes in the estimated curves would signal that the findings depend on the assumed missingness mechanism. The modest changes observed here suggest limited sensitivity within the scenarios examined; however, this sensitivity analysis did not establish robustness to MNAR mechanisms more generally.

**FIGURE 5:**
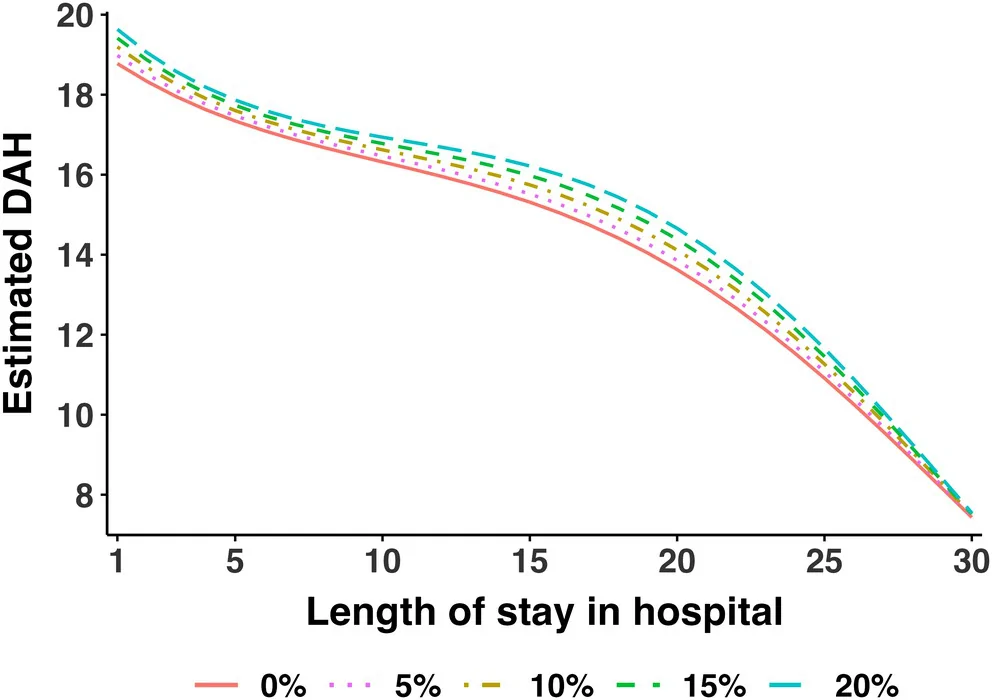
Sensitivity analysis for violation of MAR assumption. The variance of generated latent variable varies from 0% to 20% of the variance of the observed outcome.

## 7 Discussion

We have developed a doubly robust estimation dealing with a continuous treatment/exposure and missing outcomes and provided a useful practical guidance to inform the best practice of the proposed method. Both theoretical analysis and simulation studies have demonstrated the validity of our proposed method, showcasing its robustness against model mis‐specification. Applying the proposed estimator to real‐world data suggests that fracture patients with days hospital length of stay over 20 days experience substantially poorer recovery outcomes, whereas those with fewer than 20 days show a more consistent favorable recovery after discharge after controlling for baseline factors. This finding highlights the utility of hospital length of stay as an independent risk factor, beyond baseline factors, and the importance of tailored post‐acute care planning for those patients with hospital stay longer than 20 days. However, it is important to note that the causal interpretation of hospital length of stay as an intervention warrants caution, as length of stay may reflect both discharge‐management decisions [43] and the evolving clinical course during hospitalization, which may not be fully intervenable.

Building on this method, there are several promising directions that merit additional efforts.

**Machine learning.** Currently, our experiments primarily rely on parametric models to estimate nuisance components, based on the satisfactory results from the model diagnostics and performance checks (Figures 3 and 5), which have been shown to be appropriate in real‐data applications. However, machine learning methods may be preferable in settings where parametric models are believed to be mis‐specified or fail to achieve adequate performance based on diagnostic evaluations. Due to its debiased nature of doubly robust estimators [18, 19, 44], the framework also accommodates modern machine learning methods to estimate nuisance functions, such as gradient boosting, random forests, neural networks, GAMs, or Super Learner ensembles, allowing for flexible modeling of nonlinearities and high‐order interactions. Comprehensive simulation studies will be needed to further evaluate the performance under these extensions.

**Heterogeneity of exposure effects.** In addition to studying average exposure effects, it would be valuable to examine how the impact of hospital length of stay on recovery varies across key patient subgroups. For example, evaluating differential effects between males and females sub‐cohorts could offer insights for optimizing care management, as male fracture patients have shown to be associated with poorer recovery outcome [45]. However, achieving this goal would require the development of a new theoretical framework, which is beyond the scope of this paper.

**Time‐varying exposure.** Beyond hospital length of stay, many real‐world exposures vary over time, such as air pollutant concentrations. Studying the effects of aggregated or long‐term exposure to high fine particulate matter (PM2.5) levels on health outcomes is therefore essential. However, addressing this question requires the development of a new causal inference framework that can properly account for time‐varying confounding effects and exposure‐confounder feedback loop. Additional efforts will be needed in future studies.

**Missing not at random (MNAR).** In Medicare fee‐for‐service claims data, follow‐up may be truncated for several reasons, including insurance switching or death, which may trigger a MNAR mechanism. Notably, MNAR cannot generally be verified using the observed data alone and requires additional investigation [46, 47], which is beyond the scope of this paper and left for future work.

## Funding

C. Chen's work was partly supported by the National Institutes of Health (NIH) grants P30AG028747 and R01AG089377. This research was also partly supported by University of Maryland, Baltimore through a grant from the Pivot Strategic Investment Initiative. The contents are solely the responsibility of the authors and do not necessarily represent the official views of NIH or University of Maryland, Baltimore.

## Disclosure

The authors have nothing to report.

## Conflicts of Interest

The authors declare no conflicts of interest.

## Supporting information




**Data S1.** Supporting Information.

## Data Availability

The data that support the findings of this study are available on request from the corresponding author. The data are not publicly available due to privacy or ethical restrictions.
