# Supplementary material for: A Doubly Robust Approach to Evaluating the Impact of Hospital Length of Stay on Post‐Fracture Recovery in Older Adults With Cognitive Impairment
Source: Stat Med. 2026 Oct 15;45(23-24):e70768. doi: 10.1002/sim.70768 (PMC13672678; doi:10.1002/sim.70768)
Supplement: Supplementary file 1 [file SIM-45-0-s001.pdf]

28

Supplementary Materials for "A Doubly Robust Approach to Evaluating the Impact of Hospital Length of Stay on Post-Fracture Recovery in Older Adults with Cognitive Impairment"

Haoyu Ren[1], Tong Wen[2], Jason Falvey[3], Yi Huang*[1], Chixiang Chen*[2]
*Chixiang Chen. Email: chixiang.chen@som.umaryland.edu
*Yi Huang. Email: yihuang@umbc.edu

The Supplementary Material is organized as follows. Section S1describes the additional notations and assumptions mentioned in the main text. Section S2 provides the proof details for Theorem 1 in the main context. Section S3 presents additional simulation resultsRMSE, bootstrap standard errors, and coverage probabilitiesfor scenarios not shown in the main text. Section S4 introduces the numbers that we used to represent covariates and exposure in real-world data analysis.

S1 | NOTATION AND ASSUMPTION

Let N denote the total number of the sample size, d be the dimension of our spline function $\boldsymbol{\eta}$. For two sequences of random variables a_N, b_N, $N \geq 1$: $a_N \lesssim_{\mathbb{P}} b_N$ means $a_N = O_{\mathbb{P}}(b_N)$. For two sequences of numbers a_N, b_N, $N \geq 1$: $a_N \lesssim b_N$ means $a_N = O(b_N)$. Let $a \wedge b = \min\{a, b\}$, $a \vee b = \max\{a, b\}$. The ℓ_2 norm of a vector is denoted by $\|\cdot\|$, the ℓ_1 norm by $\|\cdot\|_1$, the ℓ_∞ norm by $\|\cdot\|_\infty$, and ℓ_0 by $\|\cdot\|_0$. For a random variable V, let $\|V\|_{p,q} := (\int |V|^q dP)^{1/q}$. With the $\mathbb{E}(x)$ represents the empirical measurement of expectation.

Besides the assumptions mentioned in the main text, in order to show the estimation consistency and the convergence rate, we need additional assumptions to show the convergence error rate of $\hat{\boldsymbol{\beta}}$, which have been well established and adopted by Semenova and Chernozhukov**?** .

Assumption S1 (Identification)**.** Let $Q := \mathbb{E}\big[\boldsymbol{\eta}(A)\boldsymbol{\eta}(A)^\top\big]$ denote the population covariance matrix of $\boldsymbol{\eta}(A)$. Assume there exist constants $0 < C_{\min} < C_{\max} < \infty$ such that $C_{\min} \leq \min \operatorname{eig}(Q) \leq \max \operatorname{eig}(Q) \leq C_{\max}$.

Assumption S2 (Growth Condition)**.** Let the supnorm of the basis functions be

$$\epsilon_d := \sup_{a \in \mathcal{A}} \|\boldsymbol{\eta}(A)\| = \sup_{a \in \mathcal{A}} \Big\{ \sum_{j=1}^{d} \eta_j(a)^2 \Big\}^{1/2}.$$

We assume it grows sufficiently slowly: $\sqrt{\frac{\epsilon_d^2 \log N}{N}} = o(1)$.

Define the stochastic error $U := Y^*(\xi_0) - Y^*(\tilde{\xi}_0)$, where $Y^*(\tilde{\xi}_0) = (Y - \mu_0) * \omega_0 / s_0 + \int \mu_0 dF(\mathbf{x})$, lower and upper bounds on its conditional second moment:

$$\underline{\sigma}^2 := \inf_{a \in \mathcal{A}} \mathbb{E}\big(U^2 \mid A = a\big), \qquad \overline{\sigma}^2 := \sup_{a \in \mathcal{A}} \mathbb{E}\big(U^2 \mid A = a\big).$$

Assumption S3 (Misspecification Error)**.** There exists a sequence of finite constants ℓ_d, r_d such that the norms of $r_g = Y^*(\tilde{\xi}_0) - \boldsymbol{\eta}^\top \boldsymbol{\beta}_0$ are controlled as follows:

$$\|r_g\|_{P,2} := \Big\{ \int r_g(a)^2 \, dP(a) \Big\}^{1/2} \lesssim r_d, \qquad \|r_g\|_{P,\infty} := \sup_{a \in \mathcal{A}} \big| r_g(a) \big| \lesssim \ell_d \, r_d.$$

Assumption S4 (Error Assumption)**.** The second moment of the sampling error U conditional on $\mathbf{X}$ is bounded from above:

$$\overline{\sigma}^2 \lesssim 1.$$

We introduced a sequence of nuisance realization sets $\mathcal{T}_N$ for the nuisance parameter ξ_0. As the sample size N increases, the sets $\mathcal{T}_N$ shrink around the true value ξ_0. The shrinkage speed is captured by the statistical rates B_N and Λ_N defined below.

Assumption S5. (Small Bias Condition) There exists a sequence $\varepsilon_N = o(1)$ such that, with probability at least $1 - \varepsilon_N$, the estimator $\hat{\xi}$ belongs to the shrinking neighborhood of ξ_0, denoted by $\mathcal{T}_N$. Uniformly over $\mathcal{T}_N$ the following meansquare convergence holds:

$$B_N := \sqrt{N} \sup_{\xi \in \mathcal{T}_N} \Big\| \mathbb{E}\, \boldsymbol{\eta}(A) \{Y^*(\xi) - Y^*(\xi_0)\} \Big\| = o(1),$$

$$\Lambda_N := \sup_{\xi \in \mathcal{T}_N} \Big(\mathbb{E} \Big\| \boldsymbol{\eta}(A) \{Y^*(\xi) - Y^*(\xi_0)\} \Big\|^2 \Big)^{1/2} = o(1).$$

In particular, $\Lambda_N \lesssim \epsilon_d \, \sup_{\xi \in \mathcal{T}_N} \big[\mathbb{E}\{Y^*(\xi) - Y^*(\xi_0)\}^2 \big]^{1/2}$.

S2 | PROOF OF THEOREM 1 IN THE MAIN CONTEXT

In this section, we first present the proof of the doubly robust property of our proposed estimator: $\hat{\boldsymbol{\beta}}$ is a consistent estimator of

Let $B = (Y - \mu) * \omega/s + \int \mu \, dF(\mathbf{x})$, $m = \int \mu dF(\mathbf{x})$, and $B_0 = (Y - \mu_0)\omega_0/s_0 + \int \mu_0 \, dF(\mathbf{x})$, $m_0 = \int \mu_0 dF(\mathbf{x})$. By simple algebra, we have

$$\begin{aligned} E\big\{Y^*(\xi) - Y^*(\xi_0)|A = a\big\} &= E\left(\frac{R}{\pi}B - \frac{R-\pi}{\pi}m - \frac{R}{\pi_0}B_0 + \frac{R-\pi_0}{\pi_0}m_0\middle|A = a\right) \\ &= E\left\{\frac{R}{\pi}(B-m) + m - \frac{R}{\pi_0}(B_0 - m_0) - m_0\middle|A = a\right\} \\ &= E\left\{\frac{R}{\pi}(B-m) - \frac{R}{\pi_0}(B_0 - m_0)\middle|A = a\right\} + E(m - m_0|A = a). \end{aligned}$$

By analyzing the first component on the right hand side of the third equation above, we have

$$\begin{aligned} E\left(R\frac{B\pi_0 - m\pi_0 - B_0\pi + m_0\pi}{\pi\pi_0}\middle|A = a\right) &= E\left\{\frac{R}{\pi\pi_0}\big(B\pi_0 - B_0\pi_0 + B_0\pi_0 - B_0\pi - m\pi_0 + m_0\pi_0 - m_0\pi_0 + m_0\pi\big|A = a\big)\right\} \\ &= E\left[E\left\{\frac{R}{\pi\pi_0}\big(B\pi_0 - B_0\pi_0 + B_0\pi_0 - B_0\pi - m\pi_0 + m_0\pi_0 - m_0\pi_0 + m_0\pi\big)\middle|A = a, \mathbf{X}\right.\right. \\ &\left.\left.\middle|A = a\right\}\right] \\ &= E\left[\frac{1}{\pi}\big\{\pi_0(B - B_0) + (\pi_0 - \pi)B_0 - \pi_0(m - m_0) - m_0(\pi_0 - \pi)\big\}\middle|A = a\right] \\ &= E\left(\frac{1}{\pi}\big[\pi_0\{(B - B_0) - (m - m_0)\} + (\pi_0 - \pi)(B_0 - m_0)\big]|A = a\right). \end{aligned}$$

The observation indicator R is dealt with due to the assumption $R \perp\!\!\!\perp Y \mid \mathbf{X}, A$ and conditional expectation properties.

With the above derivation, by simple algebra, we have

$$\begin{aligned} E\{Y^*(\xi) - Y^*(\xi_0)|A = a\} &= E\left[\frac{1}{\pi}\big\{\pi_0(B - B_0) - (m - m_0)\big\} + (\pi_0 - \pi)(B_0 - m_0)|A = a\right] \\ &+ E(m - m_0|A = a) \\ &= E\left[\frac{1}{\pi}\big\{\pi_0(B - B_0) - (m - m_0)\big\} + (\pi_0 - \pi)(B_0 - m_0)|A = a\right] \\ &+ E\left\{\frac{\pi}{\pi}(m - m_0)|A = a\right\} \\ &= E\left[\frac{1}{\pi}\big\{\pi_0(B - B_0) + (\pi - \pi_0)(m - m_0) + (\pi_0 - \pi)(B_0 - m_0)\big\}|A = a\right] \\ &= E\left[\frac{1}{\pi}\big\{\pi_0(B - B_0) + (\pi - \pi_0)(m - m_0)\big\}|A = a\right]. \end{aligned}$$

To show that the above formula equals zero, it is important to evaluate $\mathbf{E}\big[B \mid A = a\big]$. Note that

$$\begin{aligned} E\big(B \mid A = a\big) &= E\left\{\frac{Y - \mu(A, \mathbf{X})}{s(A \mid \mathbf{X})/\omega(a)} + \int \mu(A, \mathbf{X}) dF(\mathbf{X}) \;\middle|\; A = a\right\} \\ &= \int \frac{\mu_0(a, \mathbf{X}) - \mu(a, \mathbf{X})}{s(a \mid \mathbf{X})/\omega(a)} dF(\mathbf{X} \mid a) + \int \mu(a, \mathbf{X}) dF(\mathbf{X}) \\ &= \int \big\{\mu_0(a, \mathbf{X}) - \mu(a, \mathbf{X})\big\} \frac{s_0(a \mid \mathbf{X})/\omega_0(a)}{s(a \mid \mathbf{X})/\omega(a)} dF(\mathbf{X}) + \int \mu(a, \mathbf{X}) dF(\mathbf{X}) \\ &\overset{if\, \mu=\mu_0}{=} \int \mu_0(a, \mathbf{X}) dF(\mathbf{X}). \\ &\overset{if\, s=s_0}{=} \int \mu_0(a, \mathbf{X}) - \mu(a, \mathbf{X}) + \mu(a, \mathbf{X}) dF(\mathbf{X}) = \int \mu_0(a, \mathbf{X}) dF(\mathbf{X}). \end{aligned}$$

The second equation holds because $p(\mathbf{x}|a) = s(a|\mathbf{x})p(\mathbf{x})/\omega(a)$, and we replace the differential part $dF(\mathbf{x}|a)$ by $dF(\mathbf{x})$**?** . Under the double robustness condition, as a result, we have $E(B|A = a) = \int \mu_0(a, \mathbf{X}) dF(\mathbf{X})$.

Similarly, we have $E\big(B_0 \mid A = a\big) = \int \mu_0(a, \mathbf{X}) dF(\mathbf{X})$. Therefore, we have $E(B - B_0|A = a) = \mathbf{0}$ if either (i) s & π or (ii) μ is correctly specified.

Moreover, it is straightforward to show that when either π or μ is correctly specified, $E\{(\pi - \pi_0)(m - m_0)|A = a\} = \mathbf{0}$. Therefore, by conditional expectation, we have $E\{\boldsymbol{\eta}(A)(Y^*(\xi) - Y^*(\xi_0)\} = \mathbf{0}$. This completes the proof that the first component of $E\{\boldsymbol{\eta}(A)(Y^*(\xi) - \boldsymbol{\eta}(A)^\top \boldsymbol{\beta}_0)\}$ equals zero: $E\{\boldsymbol{\eta}(A)(Y^*(\xi) - Y^*(\xi_0)\} = \mathbf{0}$.

Now, we deal with the second component of $E\{\boldsymbol{\eta}(A)(Y^*(\xi) - \boldsymbol{\eta}(A)^\top \boldsymbol{\beta}_0)\}$. Note that, for any a, we have $E\{Y^*(\xi_0)|A = a\} = E\{Y^a|A = a\}$, because

$$\begin{aligned}
E\{Y^*(\xi_0)|A = a\} &= E\left[E\left\{\frac{R}{\pi_0}\left(\frac{Y - \mu_0}{s_0}\omega_0 + \int \mu_0 dF(\mathbf{x})\middle|\mathbf{X}, A\right)\middle|A = a\right\}\right] \\
&= E\left(\frac{Y - \mu_0}{s_0}\omega_0 + \int \mu_0 dF(\mathbf{x})\middle|A = a\right) \\
&= E\{Y^a|A = a\}.
\end{aligned}$$

The second equation holds by the assumption $R \perp\!\!\!\perp Y \mid \mathbf{X}, A$ and conditional expectation properties. The third equation holds by following arguments in the proof of Kennedy et. al.[?]. Thus, by the definition of $\boldsymbol{\beta}_0$, which solves $E\{\boldsymbol{\eta}(A)(Y^*(\xi_0) - \boldsymbol{\eta}(A)^\top \boldsymbol{\beta}_0\} = \mathbf{0}$, and by conditional expectation properties, we have $E\{\boldsymbol{\eta}(A)(Y^*(\xi) - \boldsymbol{\eta}(A)^\top \boldsymbol{\beta}_0)\} = \mathbf{0}$. Finally, applying the Z-estimator theory (Theorem 5.9) with regularity conditions[?] completes the proof that $\hat{\boldsymbol{\beta}}$ is a consistent estimator under the double robustness condition with $\int \mu_0 dF(\mathbf{x})$ replaced by a consistent estimator.

With established doubly robust property, we next sketch the proof of convergence error rate for the resulting estimator, by following the proof strategy from Semenova and Chernozhukov[?]. Note that

$$\begin{aligned}
\|\hat{\boldsymbol{\beta}} - \boldsymbol{\beta}_0\| &= \left\|\hat{Q}^{-1}\right\|\left\|\mathbb{E}_N \boldsymbol{\eta}_i^\top Y_i^*(\hat{\xi}) - \boldsymbol{\beta}_0\right\| \\
&\leq \|\hat{Q}^{-1}\|\left\|\mathbb{E}_N \boldsymbol{\eta}_i[Y_i^*(\hat{\xi})) - Y_i^*(\xi_0)]\right\| + \|\hat{Q}^{-1}\|\left\|\mathbb{E}_N \boldsymbol{\eta}_i[Y_i^*(\xi_0)) - \eta_i^\top \boldsymbol{\beta}_0]\right\| \\
&= S_1 + \|\hat{Q}^{-1}\|\left\|\mathbb{E}_N \boldsymbol{\eta}_i[Y^*(\xi_0) - Y^*(\tilde{\xi}_0)]\right\| + \left\|\mathbb{E}_N \boldsymbol{\eta}_i[Y^*(\tilde{\xi}_0) - \boldsymbol{\eta}_i^\top \boldsymbol{\beta}_0]\right\| \\
&= S_1 + \|\hat{Q}^{-1}\|\ \|\mathbb{E}_N \boldsymbol{\eta}_i^\top U_i\| + \|\hat{Q}^{-1}\|\ \|\mathbb{E}_N \boldsymbol{\eta}_i^\top r_i\|,
\end{aligned}$$

where $\hat{\boldsymbol{\beta}} = \left\{\sum_{i=1}^N \boldsymbol{\eta}(A_i)\boldsymbol{\eta}(A_i)^\top\right\}^{-1}\left\{\sum_{i=1}^N \boldsymbol{\eta}(A_i)Y_i^*(\hat{\xi})\right\}$ and $S_1 = \|\hat{Q}^{-1}\|\left\|\mathbb{E}_N \boldsymbol{\eta}_i[Y_i^*(\hat{\xi})) - Y_i^*(\xi_0)]\right\|$. Suppose assumption S4 holds, the effect of sampling error (the second term) is bounded as

$$\|\mathbb{E}_N \boldsymbol{\eta}_i^\top U_i\| \lesssim_P \left(\mathbb{E}\left[\|\mathbb{E}_N \boldsymbol{\eta}_i^\top U_i\|^2\right]\right)^{1/2} \leq \left(\mathbb{E}\left[U_i^2\, \boldsymbol{\eta}_i^\top \boldsymbol{\eta}_i\right]/N\right)^{1/2} \lesssim \bar{\sigma}\sqrt{d/N}.$$

By assumption S3, the effect of approximation error (the third term) is bounded as

$$\left\|\mathbb{E}_N \boldsymbol{\eta}_i^\top r_i\right\| \lesssim_P \left(\mathbb{E}\left[\|\mathbb{E}_N \boldsymbol{\eta}_i^\top r_i\|^2\right]\right)^{1/2} \lesssim l_d r_d \sqrt{\frac{\mathbb{E}\|\boldsymbol{\eta}_i\|^2}{N}} = l_d r_d \sqrt{\frac{d}{N}}.$$

Alternatively, by the assumption S2 and assumption S3 the third term can also be bounded as follows:

$$\left\|\mathbb{E}_N \boldsymbol{\eta}_i^\top r_i\right\| \lesssim_P \left(\mathbb{E}\left[\|\mathbb{E}_N \boldsymbol{\eta}_i^\top r_i\|^2\right]\right)^{1/2} \leq \epsilon_d r_d \sqrt{\frac{\mathbb{E}r_i^2}{N}} = \epsilon_d r_d / \sqrt{N}$$

holds with high probability, $\|\hat{Q}^{-1}\| \leq 2\|Q^{-1}\| \leq 2/C_{\min}$ by assumption S1.

Finally, assuming that the true nuisance functions belong to the Donsker class, Lemma 1 below implies that

$$\|S_1\| \leq \|\hat{Q}^{-1}\| \left\|\mathbb{E}_N \eta_i\left[Y_i^*(\hat{\xi}) - Y_i^*(\xi_0)\right]\right\| \leq \|\hat{Q}^{-1}\| \left[B_N/\sqrt{N} + \Lambda_N/\sqrt{N}\right] = o_P(N^{-1/2}).$$

Thus, the resulting estimator $\hat{\boldsymbol{\beta}}$ is bounded as:

$$\left\|\hat{\boldsymbol{\beta}} - \boldsymbol{\beta}_0\right\| \lesssim_P \sqrt{\frac{d}{n}} + \left[\left(\sqrt{\tfrac{d}{n}}\, l_d r_d\right) \wedge \left(\epsilon_d r_d/\sqrt{n}\right)\right],$$

which completes the proof of Theorem 1 in the main context.

Lemma 1 (No Effect of First Stage Error)**.** Suppose Assumption S5 holds. We have

$$\sqrt{N}\left\|\mathbb{E}_N\, \eta_i\left[Y_i^*(\hat{\xi}) - Y_i^*(\xi_0)\right]\right\| = O_{\mathbb{P}}(B_N + \Lambda_N) = o(1). \tag{3}$$

This lemma restates Lemma 3.1 from Semenova and Chernozhukov[?], extended to incorporate nuisance parameters arising from the missing data model. The proof follows a similar strategy and is therefore omitted.

S3 | SIMULATION RELATED

As mentioned in section 5 of the main text, we generate five covariates coming from a multivariate normal distribution with the covariance matrix $\mathbf{\Sigma}$ and another five covariates coming from the multivariate binomial distribution with variance matrix $\boldsymbol{\rho}$, where

$$\mathbf{\Sigma} = \begin{bmatrix} 1.00 & 0.17 & -0.12 & 0.24 & 0.05 \\ 0.17 & 1.00 & -0.12 & 0.22 & -0.44 \\ -0.12 & -0.12 & 1.00 & 0.21 & 0.26 \\ 0.24 & 0.22 & 0.21 & 1.00 & -0.06 \\ 0.05 & -0.44 & 0.26 & -0.06 & 1.00 \end{bmatrix} \qquad \boldsymbol{\rho} = \begin{bmatrix} 1.00 & -0.15 & 0.02 & 0.21 & 0.04 \\ -0.15 & 1.00 & 0.09 & -0.05 & -0.04 \\ 0.02 & 0.09 & 1.00 & 0.15 & 0.26 \\ 0.21 & -0.05 & 0.15 & 1.00 & 0.08 \\ 0.04 & -0.04 & 0.26 & 0.08 & 1.00 \end{bmatrix}$$

Moreover, we generated the true values $\boldsymbol{\beta}_0$ by projecting the estimated integral of the true conditional mean $\boldsymbol{\mu}$ using leave-one-out method[?] onto a spline expansion of A, under a cohort with a very large sample size.

The results based on the sample size of 1000 are summarized in Figure S1 and Figure S2. The overall pattern and conclusion did not change as sample size varied. But with a smaller sample size, the overall RMSE became larger than the RMSE under sample size of 3000. Moreover, our proposed method exhibited a notably smaller variance than the competing approaches. We also computed bootstrap standard errors across multiple scenarios; the resulting coverage probabilities (for nominal 95% intervals) consistently fell between 93% and 97%, regardless of sample size. Moreover, Tables S1, S2, and S3 show that the bootstrap standard errors closely tracked the Monte Carlo standard deviations. These results demonstrate the validity of bootstrap inference in practice.

Moreover, we conducted two sensitivity analyses. One was to evaluate the performance under 2000 Monte Carlo iterations. As the results based on 500 Monte Carlo iterations (Figure S2) were very similar to those based on 2000 iterations (Figure S3), 500 Monte Carlo runs appeared sufficient to provide stable estimates of bias and coverage probability. The other was to evaluate the performance under 500 bootstrap samples. Notably, increasing the number of bootstrap samples from 50 (Table S3) to 500 (Table S5) produced similar inference results, suggesting that 50 bootstrap samples might be adequate for estimating standard errors and coverage probabilities.

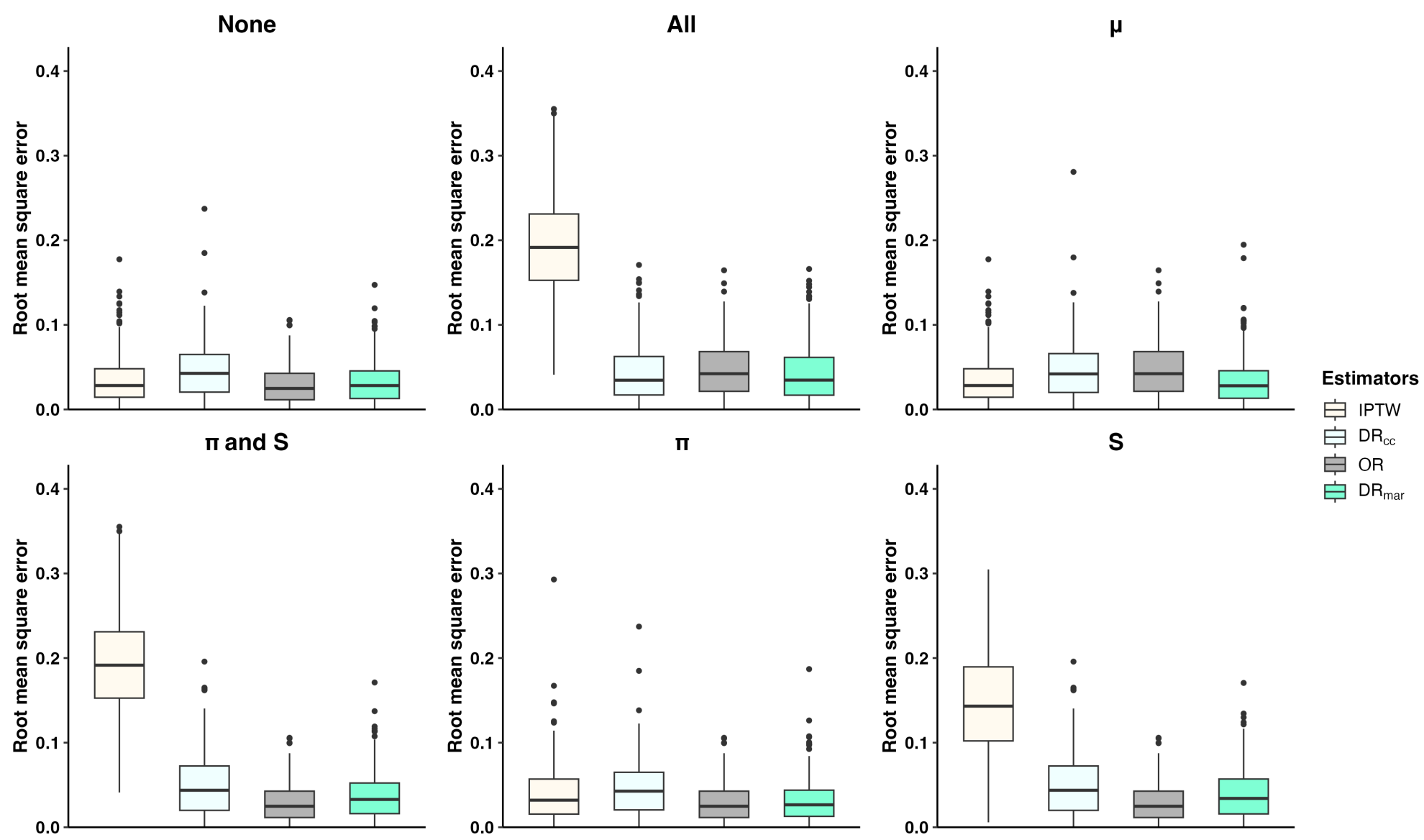

FIGURE S1 Evaluation the performance of different estimators under settings with different misspecified models, sample size of 1000, and missing rate 20% with 500 Monte-Carlo.

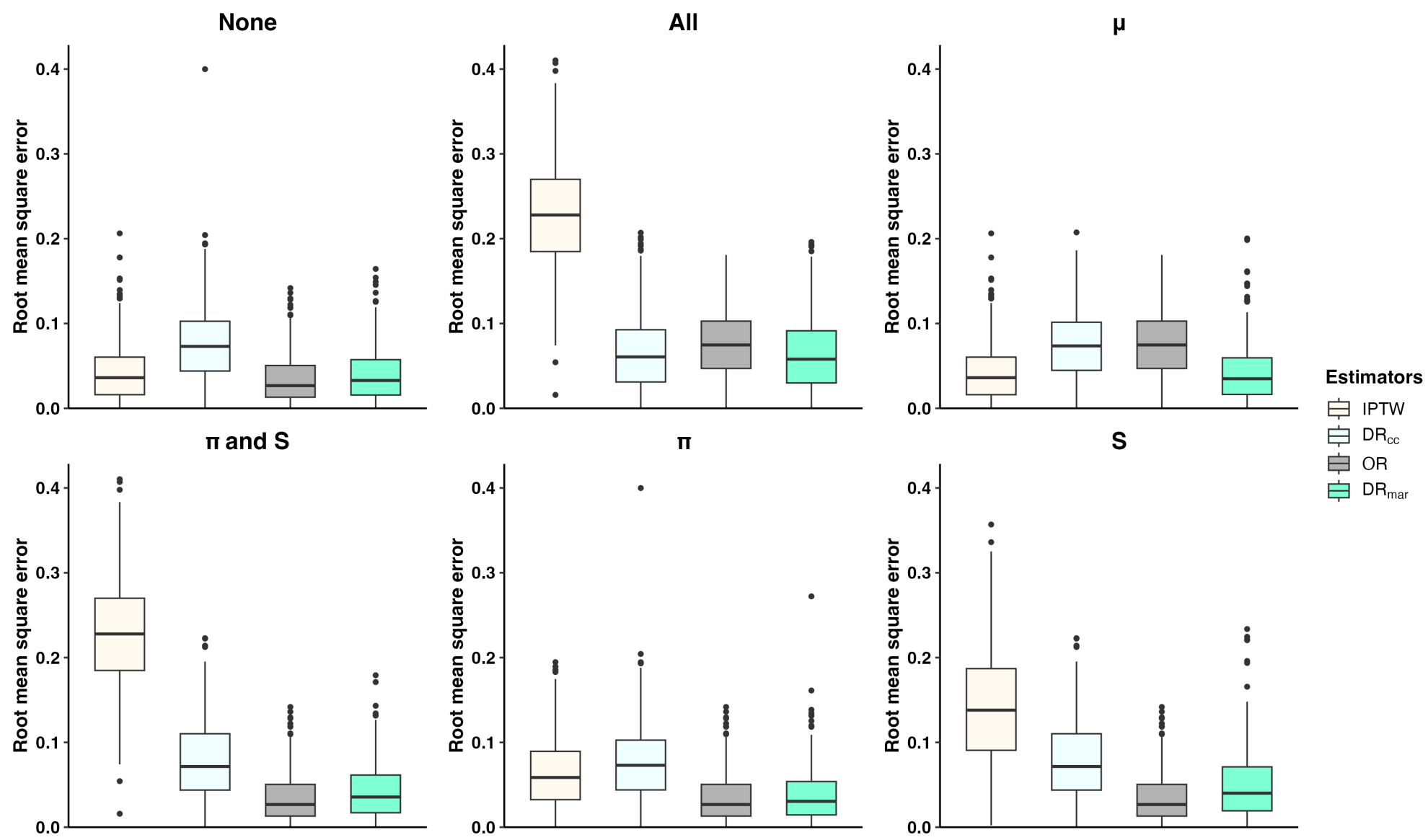

FIGURE S2 Evaluation the performance of different estimators under settings with different mis-specified models, sample size of 1000, and missing rate 40% with 500 Monte-Carlo.

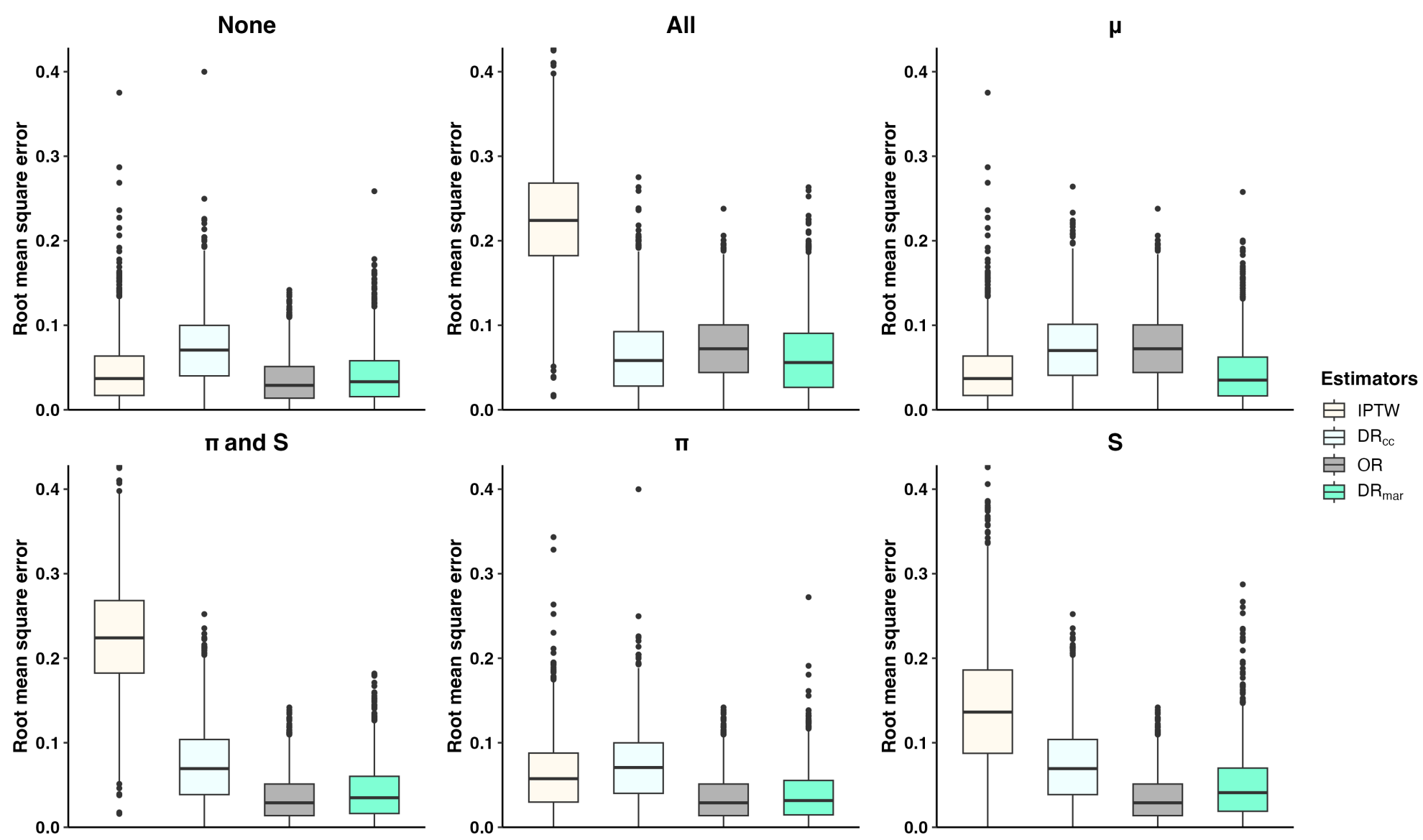

FIGURE S3 Evaluation the performance of different estimators under settings with different mis-specified models, sample size of 1000, and missing rate 40% with 2000 Monte-Carlo.

S4 | EXTRA INFORMATION FROM REAL DATA ANALYSIS

For concise illustration, we used numbers to replace the full variable names, with detailed information summarized in the following chart S4.

	None		μ		S		π		π & S	
	BSE(MCSD)	CP	BSE(MCSD)	CP	BSE(MCSD)	CP	BSE(MCSD)	CP	BSE(MCSD)	CP
β_1	18.0(20.5)	95	21.0(25.1)	94	29.7(31.1)	95	17.6(20.8)	94	28.0(29.4)	94
β_2	28.9(31.6)	95	33.5(38.4)	95	41.8(43.1)	95	28.0(31.7)	96	39.8(41.4)	94
β_3	29.3(33.3)	94	32.7(38.9)	95	41.2(42.7)	95	28.3(32.7)	95	39.5(41.0)	94
β_4	18.6(22.6)	95	19.7(25.6)	93	28.6(29.1)	94	18.1(21.8)	95	27.5(27.8)	94

TABLE S1 Comparison of bootstrap standard error (BSE), Monte Carlo standard deviation (MCSD), and coverage probability (CP) under different misspecified models when sample size is 3000 and missing rate 20% with 50 bootstraps. (All values were multiplied by 100)

	None		μ		S		π		π & S	
	BSE(MCSD)	CP	BSE(MCSD)	CP	BSE(MCSD)	CP	BSE(MCSD)	CP	BSE(MCSD)	CP
β_1	33.5(30.5)	95	43.5(41.2)	96	57.0(53.0)	96	29.9(28.7)	96	49.6(49.5)	95
β_2	51.5(44.0)	97	66.1(59.2)	96	78.4(74.0)	97	47.1(41.9)	97	70.1(69.5)	97
β_3	50.3(43.8)	97	61.7(55.1)	96	75.8(73.1)	96	47.1(42.3)	97	69.5(69.2)	95
β_4	31.2(29.4)	96	35.7(34.5)	95	52.1(51.7)	96	29.9(28.8)	96	48.5(49.6)	95

TABLE S2 Comparison of bootstrap standard error (BSE), Monte Carlo standard deviation (MCSD), and coverage probability (CP) under different misspecified models when sample size is 1000 and missing rate 20% with 50 bootstraps. (All values were multiplied by 100)

	None		μ		S		π		π & S	
	BSE(MCSD)	CP	BSE(MCSD)	CP	BSE(MCSD)	CP	BSE(MCSD)	CP	BSE(MCSD)	CP
β_1	42.9(39.1)	94	55.0(50.0)	96	75.3(78.9)	95	34.6(33.8)	94	57.0(60.2)	93
β_2	67.6(62.7)	97	83.8(77.0)	97	105.5(113.1)	95	53.2(51.0)	96	79.6(83.8)	95
β_3	65.3(64.9)	96	76.2(77.1)	96	101.1(110.9)	96	52.1(49.8)	96	77.6(79.7)	95
β_4	38.3(43.9)	95	41.5(50.9)	94	67.3(76.8)	97	32.5(32.7)	95	53.5(53.6)	96

TABLE S3 Comparison of bootstrap standard error (BSE), Monte Carlo standard deviation (MCSD), and coverage probability (CP) under different misspecified models when sample size is 1000 and missing rate 40% with 50 bootstraps.(All values were multiplied by 100)

Pseudo names	Full names of Covariates
00	Length of stay in hospital
01	Race (White / Non-white)
02	Sex (Male / Female)
03	Average DAH for 6-month before hip fracture
04	Flag indicating a teaching hospital
05	Number of days from initial dementia diagnosis to hip fracture admission date
06	Total Elixhauser Groups
07	Surgical Procedure Type (1: Open reduction/internal fixation; 2: Total hip arthroplasty)
08	Year of Admission for Hip Fracture (0 to 8)
09	Between 200 and 425 beds
10	More than 425 beds
11	Age between 76 and 84
12	Age over 85

TABLE S4 The dictionary of covariate names used in the real data analysis.

	None		μ		S		π		π & S	
	BSE(MCSD)	CP	BSE(MCSD)	CP	BSE(MCSD)	CP	BSE(MCSD)	CP	BSE(MCSD)	CP
β_1	39.0(58.3)	96	41.0(48.8)	97	72.7(115.7)	96	39.5(36.4)	96	58.6(60.9)	96
β_2	60.0(79.7)	95	63.4(74.1)	97	100.7(147.5)	95	60.1(53.7)	96	81.7(82.8)	96
β_3	58.5(70.6)	96	59.3(68.8)	96	97.6(122.8)	96	58.4(51.6)	97	79.7(79.8)	96
β_4	35.5(44.8)	96	35.8(40.0)	94	66.0(78.3)	97	36.2(32.8)	96	54.9(55.2)	95

TABLE S5 Comparison of bootstrap standard error (BSE), Monte Carlo standard deviation (MCSD), and coverage probability (CP) under different misspecified models when sample size is 1000 and missing rate 40% with 500 bootstraps. (All values were multiplied by 100)

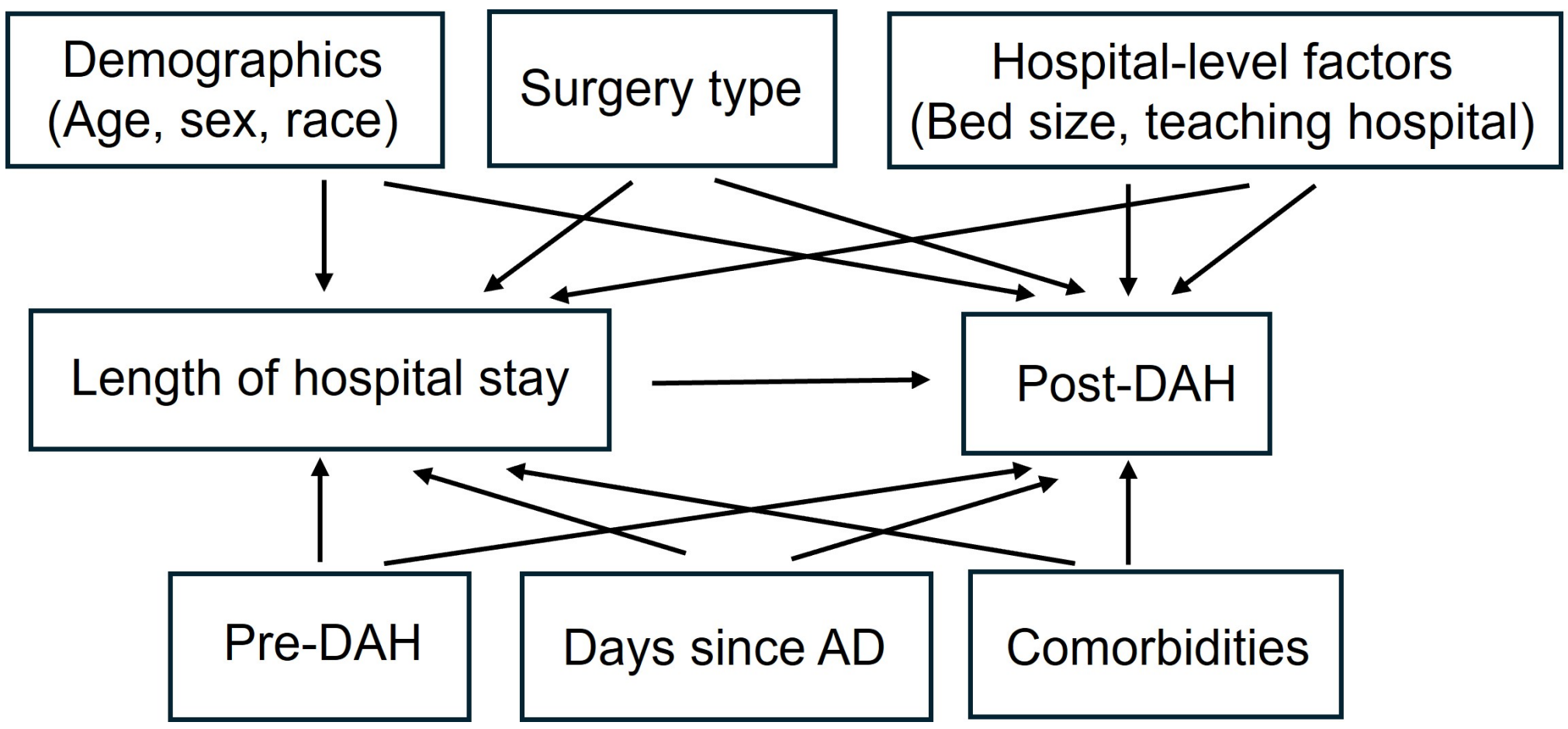

FIGURE S4 Directed acyclic graph (DAG) representing the assumed causal relationships among length of hospital stay, post-discharge days at home (Post-DAH), and measured confounders in the ADRD cohort. The confounders include demographics, surgery type, hospital-level factors, PRE-DAH, days since AD diagnosis, and comorbidities. Relationships among the confounders themselves are not displayed.

References

1. Semenova V, Chernozhukov V. Debiased Machine Learning for Conditional Average Treatment Effects and Other Causal Functions. *The Econometrics Journal* 2021; 24(2): 264–289.

2. Vaart AWvd. *Mand Z-Estimators*: 4184; Cambridge Series in Statistical and Probabilistic Mathematics. Cambridge University Press . 1998.

3. Kennedy EH, Ma Z, McHugh MD, Small DS. Non-parametric methods for doubly robust estimation of continuous treatment effects. *Journal of the Royal Statistical Society Series B* 2017; 79(4): 1229–1245.

□
